# New Microfluidic System for Electrochemical Impedance Spectroscopy Assessment of Cell Culture Performance: Design and Development of New Electrode Material

**DOI:** 10.3390/bios12070452

**Published:** 2022-06-24

**Authors:** Ayman Chmayssem, Constantin Edi Tanase, Nicolas Verplanck, Maxime Gougis, Véronique Mourier, Abdelkader Zebda, Amir M. Ghaemmaghami, Pascal Mailley

**Affiliations:** 1 University Grenoble Alpes, CEA, LETI, DTBS, F-38000 Grenoble, France; nicolas.verplanck@cea.fr (N.V.); maxime.gougis@baio-dx.com (M.G.); veronique.mourier@cea.fr (V.M.); 2 University Grenoble Alpes, TIMC-IMAG/CNRS/INSERM, UMR 5525, F-38000 Grenoble, France; abdelkader.zebda@univ-grenoble-alpes.fr; 3 Immunology & Immuno-Bioengineering Group, School of Life Sciences, Faculty of Medicine & Health Sciences, University of Nottingham, Nottingham NG7 2RD, UK; edutanase@gmail.com (C.E.T.); amir.ghaemmaghami@nottingham.ac.uk (A.M.G.)

**Keywords:** electrochemical impedance spectroscopy, cell-health monitoring, microfluidic system, carbon-IrOx electrodes

## Abstract

Electrochemical impedance spectroscopy (EIS) is widely accepted as an effective and non-destructive method to assess cell health during cell-culture. However, there is a lack of compact devices compatible with microfluidic integration and microscopy that could provide the real-time and non-invasive monitoring of cell-cultures using EIS. In this paper, we reported the design and characterization of a modular EIS testing system based on a patented technology. This device was fabricated using easily processable methodologies including screen-printing of the impedance electrodes and molding or micromachining of the cell culture chamber with an easy assembly procedure. Accordingly, to obtain processable, biocompatible and sterilizable electrode materials that lower the impact of interfacial impedance on TEER (Transepithelial electrical resistance) measurements, and to enable concomitant microscopy observations, we optimized the formulation of the electrode inks and the design of the EIS electrodes, respectively. First, electrode materials were based on carbon biocompatible inks enriched with IrOx particles to obtain low interfacial impedance electrodes approaching the performances of classical non-biocompatible Ag/AgCl second-species electrodes. Secondly, we proposed three original electrode designs, which were compared to classical disk electrodes that were optically compatible with microscopy. We assessed the impact of the electrode design on the response of the impedance sensor using COMSOL Multiphysics. Finally, the performance of the impedance spectroscopy devices was assessed in vitro using human airway epithelial cell cultures.

## 1. Introduction

Preclinical risk assessments of the safety and biocompatibility of therapeutic compounds, biomaterials, airborne pollutants and pathogens are required before their clinical validation and approval. Currently, most biocompatibility assays are usually evaluated using microscopy and endpoint tests such as, e.g., MTT, XTT, AlamaBlue that are classically used in biology labs [1,2]. However, these tests offer limited insight into the functional properties of cells. This led to the development of complementary techniques for the biocompatibility testing of biomaterials that could provide more information on the overall health of cells and tissue [3,4,5]. However, these tests could be prohibitively complicated and expensive for screening a large number of conditions. Measuring cell impedance (e.g., using transepithelial electrical resistance (TEER) sensing [6,7,8]) has emerged as a useful surrogate for determining the functional integrity of different cells and tissues, and as such is widely accepted as an effective and non-destructive method to investigate biocompatibility [9,10,11,12]; however, no automated system for this kind of measurements was reported until now.

Animal models are also routinely used for risk assessment and biocompatibility testing; however, such models are expensive, laborious and not entirely extrapolated to humans, with ethical concerns and low throughput capabilities [13,14]. Therefore, there is significant interest in identifying new in vitro models and testing platforms to assess the risk assessment of various insults [15,16]. Throughout the breathing cycle, the lungs are commonly exposed to insults from various sources (e.g., airborne pollutants and pathogens) with potentially destructive effects on the airway epithelial cells [17,18]. During and after such exposure, the lung epithelium undergoes various airway-remodeling processes such as shedding and the derangement of epithelial cells, alteration in extracellular matrix (ECM) deposition, airflow obstruction and hyper-responsiveness [19,20].

The interaction between epithelium and external insults triggers a series of intracellular signaling cascades [21]. These biological processes may modulate the cell microenvironment culminating in changes in tissue homeostasis [22]. Previous studies have shown that cytokine secretion can be activated via specific pathways triggered by exposure to an insult [23,24]. Variations in cytokine production could in turn disturb the epithelial barrier function by influencing the function and structure of the intercellular tight junctions (TJs) as well as epithelial cell health.

As a reliable surrogate for barrier tissues integrity, it is crucial to understand the dynamics of the TEER which can also lead to the understanding of various signaling pathways. Thus, the real-time monitoring of this parameter could provide critical insight into the cell environment and also assist in the development of better in vitro testing models including organ-on-a-chip platforms.

In this work, we reported the design and development of a testing system based on a patented technology using integrated impedance sensors [25]. Here, we reported the development of a new and miniaturized design of embedded impedance sensors for risk assessments of various insults. We described the fabrication process of the monitoring system, and its integration procedure into a dedicated microfluidic cell-culture chamber. We also assessed the utility of our electrode material with different characterization techniques. In the second part of this study, we characterized (by COMSOL Multiphysics^®^) the electric field profile of the different electrodes’ design and explored their distribution in the cell-culture chamber. In the last part, we examined the changes in the barrier integrity and permeability of human airway epithelial cells in the presence of an exogenous insult in real-time. We demonstrated that this set-up provides a powerful toolkit for studying the biocompatibility of various insults/biomaterials for in vitro testing.

## 2. Materials and Methods

### 2.1. Buffers and Solutions

A stock solution of PBS (Phosphate-Buffered Saline; 10× concentrate) was purchased from Sigma Aldrich and utilized for the preparation of the working solution (PBS 1× pH 7.4). Deionized water was obtained in our laboratory by an appropriate production system (Millipore). Other chemicals used in this study were of analytical grade.

### 2.2. Preparation of Electrode Material

A specific formulation of a carbon-based ink was prepared as a sensitive layer for the impedance sensors. This homemade paste was prepared as described elsewhere [26]. The instruction for the process is as follows: Add 1 g of iridium oxide powder (IrOx.2H_2_O 99.99%, purchased from Alfa Aesar^TM^, Premion^TM^) in a mortar and grind it finely for 2 min with a pestle. Add 50 g of BQ242 carbon-based ink (purchased from Gwent electronic materials Inc.) to the IrOx powder and mix it using a spatula for another 2 min. Then, shake the obtained paste with a digital roller shaker (IKA^®^ Roller 10 digital) for 12 h at 40 rpm in order to obtain a homogeneous, consistent and smooth paste. Finally, use the obtained paste for the preparation of electrodes by screen-printing.

### 2.3. Cytotoxicity Analysis

Cytotoxicity assays were performed using NIH3T3 cells for the evaluation of the biocompatibility of electrode materials as described elsewhere [27]. Briefly, cells were seeded in a 12-well culture plate at 5 × 10^4^ cells/well and incubated for 24 h at 37 °C. The electrode materials were manufactured using two different techniques, namely direct contact and release. At predetermined time points (2 and 4 h), the cells were incubated at 37 °C, 5% CO_2_ for 2–4 h with a stable tetrazolium salt reagent (WST-1, CELLPRO-RO Roche), following the manufacturer’s protocol. Three samples per condition were analyzed. After this incubation period, WST-1 was cleaved by a complex cellular mechanism to produce formazan dye that was quantitated with a scanning multi-well spectrophotometer (ELISA reader). The measured absorbance at 450 nm directly correlated with the number of viable cells. Values of cell viability (in %) represent the average absorbency (*n* = 3) ± SEM, * *p* < 0.05 relative to positive control cultures (without any contact with any materials). Negative controls correspond to the cultures where cells are exposed to hydrogen peroxide (H_2_O_2_, 10 mM), in which no cell growth and proliferation is expected (dead cells control).

### 2.4. Device Design and Fabrication

**Figure 1:**
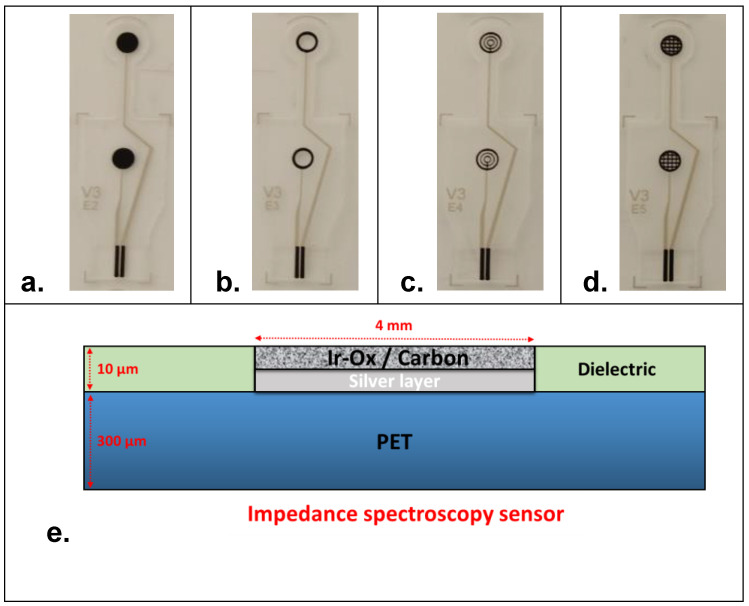
Design of the electrochemical impedance spectroscopy sensors: (**a**) planar disk-electrodes, (**b**) ring-electrodes, (**c**) multi-ring-electrodes and (**d**) grid-electrodes. (**e**) Schematic representation in sectional view of the different electrodes layers. Thickness of the screen-printed layers is given as an approximation according to the meshing of the screen and the printing parameters.

Impedance measurement of cells using TEER sensing is widely accepted as an efficient way to examine cell health. However, it is of crucial importance to many studies in cell biology to monitor cells by microscopy as it can help in understanding the dynamics of biological processes [28]. Therefore, fixing the system requirements including design aspects of electrode materials is of high importance. It is highly desirable to implement electrodes that are optically compatible with the integration of a mini-microscope in the final prototype. Indeed, this aspect can be achieved in two different ways: (1) by using transparent electrode materials (e.g., ITO electrodes [29]) or (2) by using specific designs that permit observation through the cell culture chamber for image analysis during cell-culture. A second essential condition for the employment of TEER system is the use of biocompatible materials for the development of electrodes. Indeed, most TEER studies monitor cell cultures that grow and attach directly to the surfaces of electrodes [30,31,32]. According to these primary requirements, impedance spectroscopy sensors were designed as shown in Figure 1. Four electrode designs were proposed, namely planar disk-electrodes (Figure 1a), ring-electrodes (Figure 1b), multi-ring-electrodes (Figure 1c) and grid-electrodes (Figure 1d). Indeed, each sensor design was made of two symmetrical carbon-based electrodes in the view of integration at the top and at the bottom of the cell-culture chamber. The bottom electrode served as the cell attachment and growing surface for the cells (seeding electrode). Except the first design (planar disk), all the other sensors were optically compatible with an integrated mini-microscope for the final system used.

In terms of electrode size, all the electrode designs had an external diameter of 4 mm (Figure S1). PET (Poly-Ethylene Terephthalate) sheets of a total thickness of 300 µm (±30 µm) were used as flexible material for the fabrication of electrodes using screen-printing technology. PET sheets were composed of two individual PET layers of 125 µm each (optical quality, ref. CT5) in which an adhesion layer of 50 µm (from 3 M, ref. 8212) was used as optically transparent tape to seal the two PET sheets. The transparency opening of the design of the different sensors was estimated at approx. 65%, 60% and 45%, respectively, for the ring-electrodes, the multi-ring-electrodes and the grid-electrodes. Tracks and electrodes were made of a silver layer (ref. 65001-5105). Then, a specific biocompatible formulation of carbon ink (purchased from Gwent material, BQ242) was blinded with iridium oxide powder and printed at the surface of all the electrodes according to the desired sensor design. A transparent dielectric film (ref. 65001-5309) covered the conductive tracks, except for the connecting pads and the working electrode surfaces (Figure 1e). Indeed, the aforementioned PET sheet contained 40 sensors (10 sensors per line) that were cut using a cutting machine (Mimaki, ref. CFL-605RT) for individual use (Figure S2).

Screen-printer EKRA E4 was utilized for the printing of the sensing layer using the IrOx-modified carbon ink under clean room conditions. Screen-printing (aluminum frame) type-20 with poly-esterfabric 150-034 × 22.5°, FL-260, 3–7 µm EOM (from KOENEN GmbH) was used in this study to print carbon-based inks. Lab oven (Memmert UM100) was used after screen-printing for ink/paste drying. Carbon-based inks were cured for 3–5 min at 130 °C.

**Figure 2:**
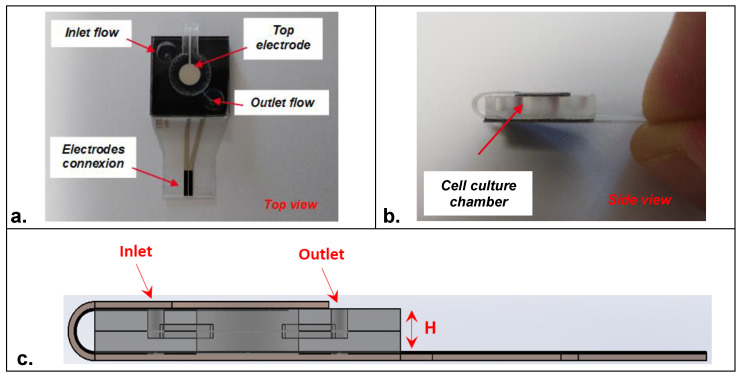
Design of the embedded microfluidic cell-culture chamber integrating the impedance sensors in (**a**) top view and (**b**) side view. (**c**) Schematic representation in section view of the assembled impedance sensors (H represents the depth of the cell-culture chamber).

The fabricated sensors were then mounted, according to an original design, in a polymer-based microfluidic cell-culture chamber made of CoC material (Cyclic olefin copolymer) (Figure 2a,b). This homemade chamber of 15 × 15 mm was designed to contain a cavity of 5 mm in which a microfluidic channel was microfabricated to function under flow rate conditions. The depth of the microfluidic chamber (H) was 1 mm, leading to a volume of around 20 μL. However, the chamber volume can be adapted to host a higher or lower volume of cell-culture medium depending on the application (Figure 2c). A double-sided pre-cut tape was utilized for system assembly (one electrode at the top and one electrode at the bottom of the cell-culture chamber). First, the part of the sensors containing the bottom electrode was mounted; then, the tab containing the top electrode was folded and mounted (Figure S3). Herein, both electrodes were brought together with the closure of the cell-culture chamber. Mini Luer-Lock connectors purchased from Microfluidic-ChipShop (Fluidic 263–10000080) were used to move the fluid through the inlet position of the cell-culture chamber.

### 2.5. Cell Culture and Seeding

Human bronchial epithelial cell lines Calu-3 (ATCC^®^ HTB-55™) were obtained from ATCC (LGC Standards, Teddington, UK) and maintained in Eagle’s Minimum Essential Medium-EMEM (Thermo Fisher Scientific, Altrincham, UK), supplemented with 10% fetal bovine serum (Sigma-Aldrich, Gillingham, UK), 1% non-essential amino acids (Gibco, UK), 1 mM sodium pyruvate (Gibco, Cambridge, UK), 1% Glutamax (Gibco, UK) and 1% penicillin/streptomycin (Sigma-Aldrich, UK) and cultured at 37 °C, 5% CO_2_ and 90% relative humidity [33]. Culture medium was changed three times per week and cell cultures were examined microscopically in order to check for any changes in viability or morphology. When they reached about 80–90%, confluence cells were passaged or used for experiments using TrypLE Express (Gibco, UK).

The devices were sterilized under UV for 15 min using a Benchmark UV-Clave UltraViolet Chamber, and prior to cell culture a fibronectin coating step (10 µg/mL) was performed for 30 min at 37 °C. Following this step, the devices were washed with PBS and the cells were seeded on the bottom electrode (seeding electrode) at 5 × 10^5^ cells/cm^−2^ and kept under static conditions for 24 h. Subsequently, a flow rate of 7 µL/min was started using Watson-Marlow Peristaltic Pump and continued for the entire duration of the experiment (6 days). The perfused system was maintained in a closed circuit. On day 5, a solution of sodium dodecyl sulfate (SDS) was added to the cell culture reservoirs at a final concentration of 0.5 mM.

### 2.6. Electrical Impedance Measurements

Measurements of electrical impedance spectroscopy (EIS) were performed at predetermined time points. Multichannel potentiostat (VPM-300) purchased from BioLogic Science Instruments (Seyssinet-Pariset, France) was used to characterize the different designs of impedance sensors. EmStat Pico module (development kit) purchased from PalmSens was used for the measurement of electrochemical signals during the cell cultures. PSTrace software (V5.8.1) was employed for the electrochemical data processing (frequency scanning range of 50 kHz–5 Hz). For electrode materials’ characterization, a classical three-electrodes cell (Ag/AgCl reference and carbon counter electrodes) was used. All other EIS experiments were conducted using the two-electrodes set-up previously described.

## 3. Results and Discussion

### 3.1. Characterization of Impedance Sensor and Selection of the Electrode Material

**Figure 3:**
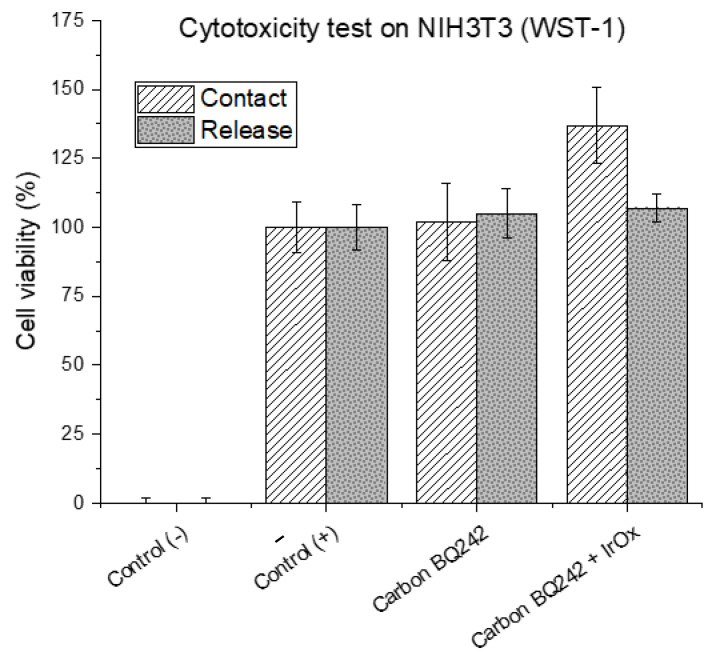
Cytotoxicity testing on NIH3T3 cells (WST-1) of electrode’s materials (carbon BQ242 and carbon BQ242 + IrOx 2 wt%). Values of cell viability (in %) represent the average absorbency (*n* = 3) ± SEM, relative to positive controls (+) cultures (without any contact with any materials). Negative controls (−) correspond to the cultures where cells are exposed to hydrogen peroxide (H_2_O_2_, 10 mM), in which no cell growth and proliferation is expected (dead cells control).

**Figure 4:**
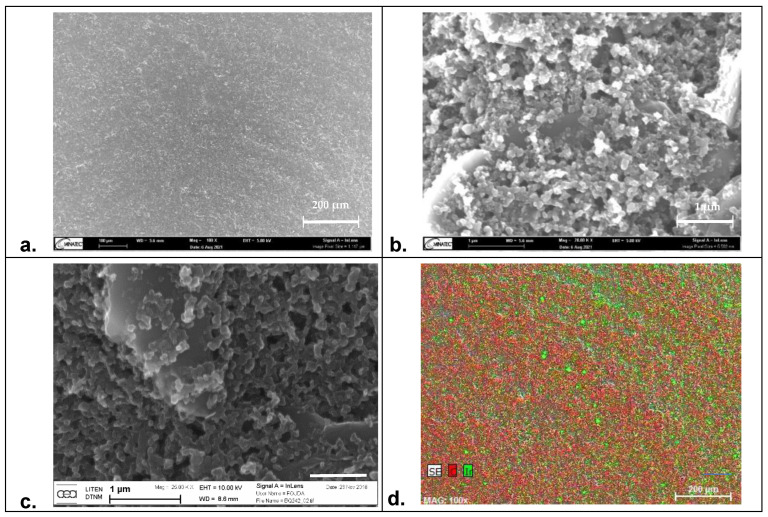
Surface morphology of carbon-based electrodes. Characterization by scanning electron microscopy (SEM) at 5 kV of the modified-carbon electrode (carbon BQ242 + IrOx 2 wt% electrode) at different magnification level of (**a**) 100× and (**b**) 20k× and (**c**) the unmodified carbon electrode at 25k×. (**d**) Electron dispersive X-ray (EDX) mapping analysis of: carbon (in red) and iridium (in green) for the modified-carbon electrode.

EIS is a commonly used technique to characterize cell health in general and for barrier tissues integrity in particular. Indeed, the materials used for the electrode fabrication have to present high biocompatibility and should overcome measurement limitations associated with the impedance of the electrochemical interface that may hinder biological events due to polarization effects. In the literature, commonly used materials for EIS and TEER measurements are gold and carbon [34,35]. If they fulfill the former requirement of biocompatibility, these materials are fully polarizable and thus present high cut-off frequencies. On the other hand, silver/silver chloride electrodes exhibit low interfacial impedance but poor biocompatibility. To combine both properties, IrOx appears as a suitable material [36,37]. To demonstrate this combination, we formulated modified-carbon inks by mixing the BQ242 carbon ink with grinded IrOx microparticles (2 and 5 wt% IrOx in carbon ink). Indeed, cytotoxicity of the carbon-based ink was evaluated on NIH3T3 cells (WST-1) using different techniques (by direct contact and by release). In addition, the cytotoxicity of the ink formulation containing the mixture of carbon ink with IrOx particles was also evaluated (Figure 3). The results reveal high cell viability that reflects the biocompatibility of the selected materials for the fabrication of the electrodes. Then, the surface morphology of the prepared electrodes (pristine BQ242 2 wt% IrOx in carbon ink) was characterized by scanning electron microscopy (SEM) analysis and electron dispersive X-ray (EDX) analysis (Figure 4).

Figure 4a,b show the recorded SEM images for the modified-carbon ink at different magnification levels of 100× and 20k×, respectively. Compared to the recorded images of the unmodified-carbon ink (Figure 4c), both surfaces show regular shapes with an unaffected morphology by the presence of IrOx particles. No cracks were observed on the electrode’s surfaces. Some aggregates of carbon particles were shown in both cases, whereas the particle size was around ~1 µm. Concomitantly, the mapping of carbon (in red) and iridium (in green) elements by EDX analysis (Figure 4d) shows that IrOx particles were uniformly distributed on the electrode’s surface. This reflects the homogeneity of the prepared paste and the high accessibility of IrOx particles on the surface. Based on this evaluation, the registered images demonstrate that the addition of IrOx particles does not change the microstructure of the electrode; however, it helps to maintain standard morphology with an enhanced interfacial behavior of the electrode.

**Figure 5:**
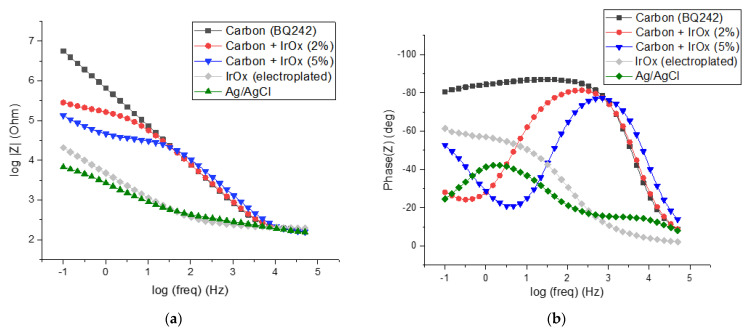
Characterization of different electrodes materials by impedance spectroscopy measurements in PBS (1X), frequency scanning range of 50 kHz–100 mHz: (**a**) impedance spectra (Bode plots) and (**b**) phase diagram of the bode plots.

No morphological evolution was demonstrated by the addition of IrOx particles. Thus, we investigated with EIS the comportment of IrOx-based electrode materials with carbon and Ag/AgCl-printed electrodes. More particularly, IrOx-carbon ink blends (2 and 5 wt% IrOx in carbon ink) were compared to Ag/AgCl and to electrodeposited IrOx electrodes to understand the benefits of IrOx implementation on TEER sensing. Figure 5 displays the impedance spectra (Bode plots) of the different electrode materials.

As expected, the Ag/AgCl electrode presented low interfacial impedance as well as a low cut-off frequency (around 100 Hz, Figure 5a). Moreover, the phase diagram of the bode plots shows a phase shift angle of lower than 40 degrees which indicates low capacitive behavior and a high availability of chloride ions ([Cl^−^] ≈ 0.014 M, Figure 5b). This behavior is typical for purely non-polarizable electrodes and is due to the reversibility of the silver/silver chloride redox reaction. On the opposite side, carbon-based electrode (BQ242) exhibits a higher cut-off frequency (around 1 kHz). As expected, the impedance of carbon materials at high frequencies (>cut-off frequency) that address the resistance of the electrolyte was similar to that of Ag/AgCl [38,39]. Down to 10 kHz, the impedance module decreased linearly with the log of frequency taking values that exceed the Ag/AgCl impedance by 2 to 3 orders of magnitude. Concomitantly, the phase of the carbon electrode reached an angle of 90° which is consistent with purely capacitive behavior. This comportment typically corresponds to a purely polarizable electrode.

As already stated, IrOx could be a material of choice for interfacing cell cultures to measure their temporal behavior. The impedance spectrum recorded for the electroplated IrOx electrode demonstrated a non-polarizable profile approaching the one of Ag/AgCl (cut-off frequency around 100 Hz). However, the IrOx phase diagram shows slightly higher phase shift angles that could be associated with the lower reversibility of the redox system. This lower reversibility could be associated both with the kinetic of the exchange of OH^−^ ions in the IrOx matrix as well as the low concentration of hydroxide ions in the solution (pH 7.4) [40]. Taking into account the potentialities of IrOx material on TEER measurements and our strategy dealing with the selection of highly processable materials (involving printing technologies), we used IrOx particles as the additive in the carbon inks to decrease their interfacial impedance.

A sensitive reduction in impedance was observed for the carbon electrodes blinded with the IrOx particles (2 and 5 wt% IrOx in carbon ink, Figure 5a) at low frequencies. As a results, the interfacial impedance of electrodes at low frequencies (down to 10 and 100 Hz, respectively for 2 and 5 wt% IrOx) decreased with the increasing amount of IrOx particles. Conversely, for the higher frequencies, the impedance of the modified carbon electrodes was dominated by the carbon material. Interestingly, the addition of IrOx in the carbon ink allows for a decreased phase at low frequencies. This may help to interpret more finely the cell culture behavior in terms of resistivity, capacitive comportment and permeability since the impedance evolution is no longer dominated by the electrochemical interfacial behavior.

### 3.2. Modeling and Selection of the Electrode Design for Cell-Culture Assessment

In order to understand the impact of the different electrode designs, it was important to model the electric field lines and to explore their distribution in the cell-culture chamber. To do so, COMSOL Multiphysics^®^ software (version 6.0), that provides simulations of the electric field distribution using the finite element method [41,42], was employed. First, the geometry of the different electrodes’ design was drawn and imported into the software. The model was constructed by means of the use of the following three domains: (1) a 1st layer domain containing a boundary of the bottom electrode, (2) a 2nd layer domain containing a boundary of the top electrode and (3) a culture chamber domain of cylindrical form (diameter of 5 mm) that geometrically separates the domains 1 and 2. It is important here to highlight that both electrodes (top and bottom) are symmetrical, while one electrode is arbitrary selected as a terminal and the second one as the ground electrode. By default, the latter corresponds to the top electrode. Indeed, the electrostatics (es) physic module was employed in our model in which we defined fixed potential boundary conditions (0 V for the ground electrode and +10 mV for the pre-defined terminal electrode). The mesh of elements was kept by default (free tetrahedral of normal size); however, a very thin element size was selected for the construction of the mesh of electrode boundaries. In our model, we assumed the presence of a large quantity of supporting electrolytes. Numerically, this is reflected by the selection of high conductivity for the medium inside the culture chamber domain (σ = 1.5 S/m). In other words, our approach consists of replicating the working conditions, especially in terms of physiological media conductivity. Under these conditions, the resistance of the solution is sufficiently low to ensure that the difference in the behavior of electrical fields is specifically associated with the differences in the design of electrodes. A frequency domain (5 Hz–50 kHz) was then selected to run the simulation study.

**Figure 6:**
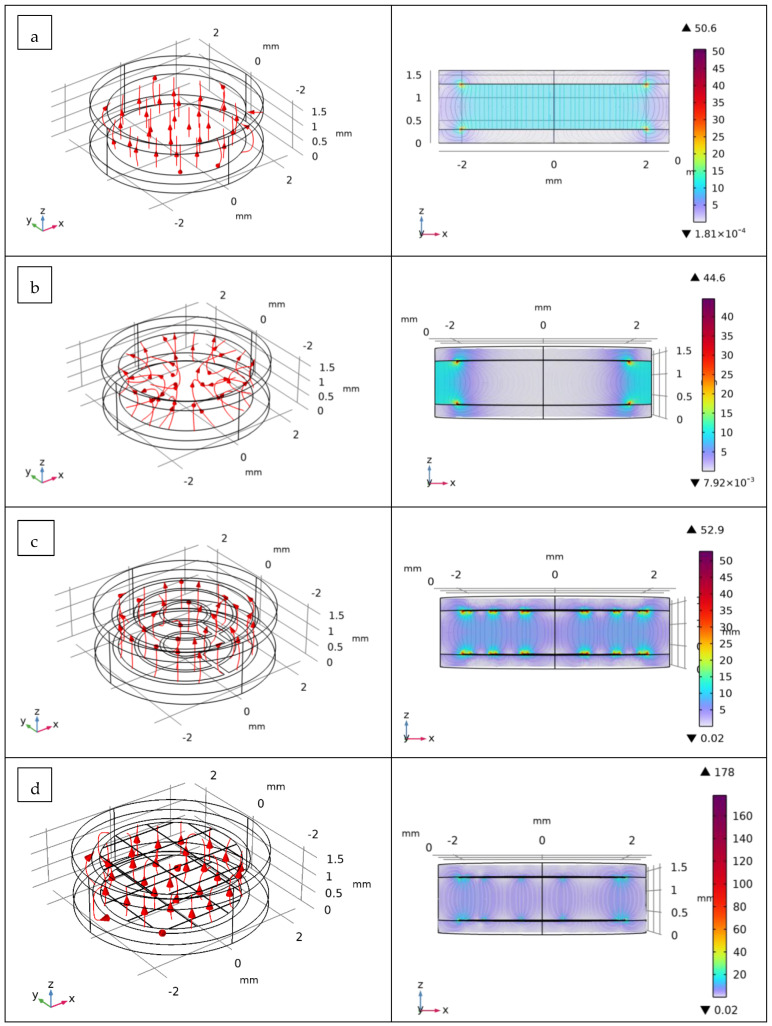
Simulation by COMSOL Multiphysics^®^ of the behavior of electric field (in V/m) inside the cell-culture chamber using the different design of impedance sensors: (**a**) planar disk-electrodes, (**b**) ring-electrodes, (**c**) multi-ring-electrodes and (**d**) grid-electrodes. The focus regarding the electric field lines in the 2D representation (section view, xz plan) is set at the middle of the chamber.

Figure 6 shows a 3D model with an xz plane (section view) of the simulation results of electrical fields (in V/m) in the cell-culture chamber using the different designs of impedance sensors. The distribution of current densities on the electrode surfaces was also investigated (Figure S4). As illustrated, for the disk-electrode design, the electrical field lines were uniformly distributed and homogenously covered the surface of the culture chamber. The behavior of this typical electrode design is expected because of the geometry of the electrodes that cover almost the whole surface of the culture chamber domain. The current densities of these electrodes were located and concentrated near the electrodes’ edge.

On the other hand, for the ring-electrode design, the electrical field lines were located between the two rings. This configuration does not allow for the accurate performance of the impedance characterization of the cells located in the middle of the culture chamber. Conversely, the use of the multi-ring-electrode design can obtain homogenous electrical field distribution without compromising the optical imaging of the cell culture.

For the last electrode design (grid-electrodes), the distribution of electrical field lines virtually covered the surface of the culture chamber; however, the values of current densities were relatively low. It is important to indicate that the latest two electrode-design EIS sensors (multi-rings-electrodes and grid-electrodes) are particularly interesting because of their compatibility with the use of the mini-microscope (high transparency opening, see Section 2.4) while maintaining a homogenous electrical field distribution in the culture chamber that accurately monitors, using EIS measurements, the cell growth in the cell-culture chamber.

### 3.3. Biological Application during Culture

To assess the performance of the impedance-measurement devices, we examined the changes in the barrier integrity of epithelial cells directly cultured within the culture chamber in real-time. The barrier integrity was assessed via impedance-spectroscopy measurements to obtain its TEER (as a surrogate readout for barrier integrity) for the entire period of the experiments. In these conditions, the cells attach, spread, and proliferate on the bottom carbon-IrOx electrode inside the cell-culture chambers (seeding electrode).

Following the attachment of Calu-3 cells onto the TEER devices, impedance measurements were recorded daily. However, to mimic a stress behavior condition, a solution of SDS was added on day 5 in the cell culture reservoirs at a final concentration of 0.5 mM. Cells cultured on the ring and multi-ring electrode-based devices were examined microscopically in order to assess any changes in cell morphology (Figure S5).

**Figure 7:**
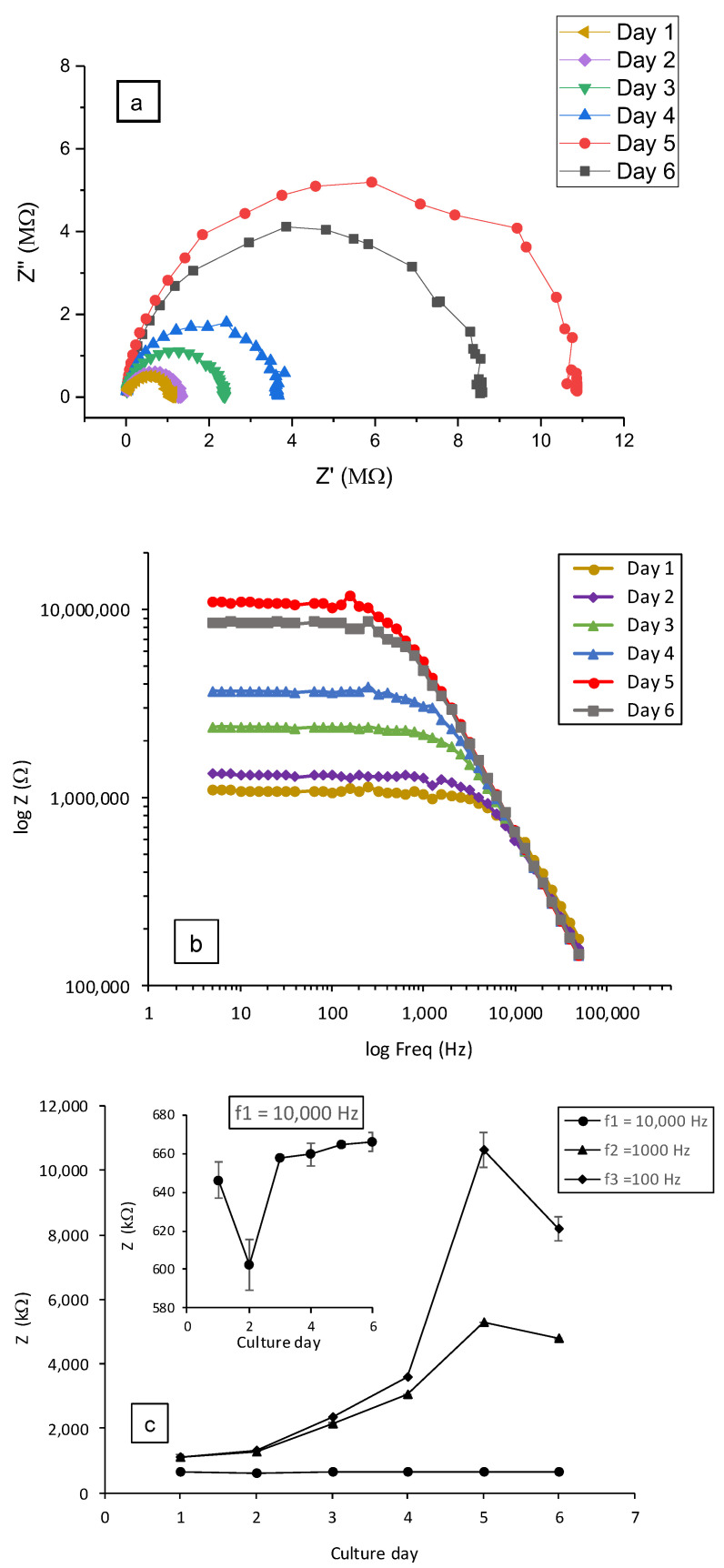
Impedance measurements (disk design, carbon BQ242 + IrOx 2 wt% electrodes) during 6 days of culture under flow rate of 7 µL·min^−1^: (**a**) Nyquist plots, (**b**) Bode plots and (**c**) impedance (Z) as function of the culture day and zoom on the plot at frequency of 10 kHz. Frequency scanning range of 50 kHz–5 Hz. Cells were exposed to SDS at a final concentration of 0.5 mM after the TEER measurement of day 5.

Figure 7 shows an example of typical data obtained from impedance spectroscopy measurements (at a frequency scanning range of 50 kHz–5 Hz) using the disk-electrodes design. As already reported, the formation of the epithelial tissue barrier is reflected by the increase in the impedance values owing to the formation of tight junctions [43,44]. In this way, Nyquist impedance plots (-Im (Z″) as a function of Re (Z′)) were traced (Figure 7a). The typical Nyquist semi-cycle radius increased with culture time until day 5. This evolution correlated well with the optical imaging of the cell growth from the attachment of dispersed cells to the formation of Calu-3 cells islands as shown in Figure S5. These results are in good correlation with impedance measurements during epithelial cell cultures reported in the literature [45,46,47]. Following the addition of SDS, on day 5, it can be seen that the Nyquist plot radius decreased (grey plot in Figure 7a) indicating the deterioration of the barrier integrity and tight junctions [43]. The same behavior can also be observed by tracing log (Z) as a function of the frequency (in logarithmic scale) (Figure 7b).

These changes of impedance (Z) were mainly detected at low frequencies as depicted in Figure 7c. This observation clearly implies that the cells grow/proliferate directly on the seeding surface (bottom electrode). For comparison, three screening frequencies were selected in our study to quantify and evaluate the change in impedance behavior at, respectively, 10 kHz, 1 kHz and 0.1 kHz (Figure 7c). At the frequency of 10 kHz, impedance decreased to only 1.03 kΩ after the addition of SDS. On the contrary, impedance decreases of 0.52 MΩ and 1.81 MΩ, respectively, at 1 kHz and 0.1 kHz were observed.

Our integrated impedance measurement device shows promise in successfully detecting damage to cells following insult/biomaterial addition and thus could act as a predictor for biomaterial risk assessment. The extent of damage according to TEER readouts and correlation to actual risk caused by the insult/biomaterial on cells would require further testing and clarification on how this can be inferred with in vivo conditions.

## 4. Conclusions

We demonstrated that the architecture of an original cell-culture chamber embedding impedance spectroscopy electrodes is compatible with cell-culture conditions and real-time TEER measurements. This device is a powerful toolkit for monitoring cell-culture growth and barrier tissues integrity. Additionally, these devices can be used to assess cell stress in a biomaterial risk assessment to complement the detection of inflammatory metabolites using multiparametric electrochemical sensing platforms [4,26]. Our strategy deals with the selection of highly processable carbon inks (which are blended with IrOx particles to decreases their interfacial impedance) with potentially low fabrication cost (involving printing technologies). The modeling by COMSOL Multiphysics software showed that the multi-rings-electrodes and grid-electrodes are of particular interest due to their compatibility with microscopy techniques (high transparency opening) while maintaining homogenous electrical field distribution in the culture chamber. This provides accurate monitoring with EIS measurements of the cell growth/damage under real-time conditions in vitro.

## Data Availability

The data that support the findings of this study are available from the corresponding authors upon reasonable request.

## Supplementary Materials

The following supporting information can be downloaded at: https://www.mdpi.com/article/10.3390/bios12070452/s1, Figure S1: Dimensions and positioning in the cell-culture chamber (in grey) of the different electrodes (in black) used for the design of impedance sensors: (design 1) planar disk-electrodes, (design 2) ring-electrodes, (design 3) multi-ring-electrodes and (design 4) grid-electrodes; Figure S2: Fabrication process of the electrical impedance spectroscopy sensors using screen-printing technology; Figure S3: Dimensions of the embedded microfluidic cell-culture chamber (in mm) integrating the impedance sensors in: (a) top view and (b) side view; Figure S4: Distribution and variation of the current density (in A/m^2^) on the electrode surfaces of: (design 1) planar disk-electrodes, (design 2) ring-electrodes, (design 3) multi-ring-electrodes and (design 4) grid-electrodes and; Figure S5: Impedance electrodes and bright field images of Calu-3 cells on (A) ring-electrodes design and (B) multi-rings electrodes after 4 days in perfused culture; magnification images highlights the cell morphology were Calu-3 cells grown as islands.
