# Supplementary material for: New Microfluidic System for Electrochemical Impedance Spectroscopy Assessment of Cell Culture Performance: Design and Development of New Electrode Material
Source: Biosensors (Basel). 2022 Jun 24;12(7):452. doi: 10.3390/bios12070452 (PMC9313146; doi:10.3390/bios12070452)
Supplement: Supplementary file 1 [file biosensors-12-00452-s001.zip › biosensors-1756729-supplementary.pdf]

Supplementary information

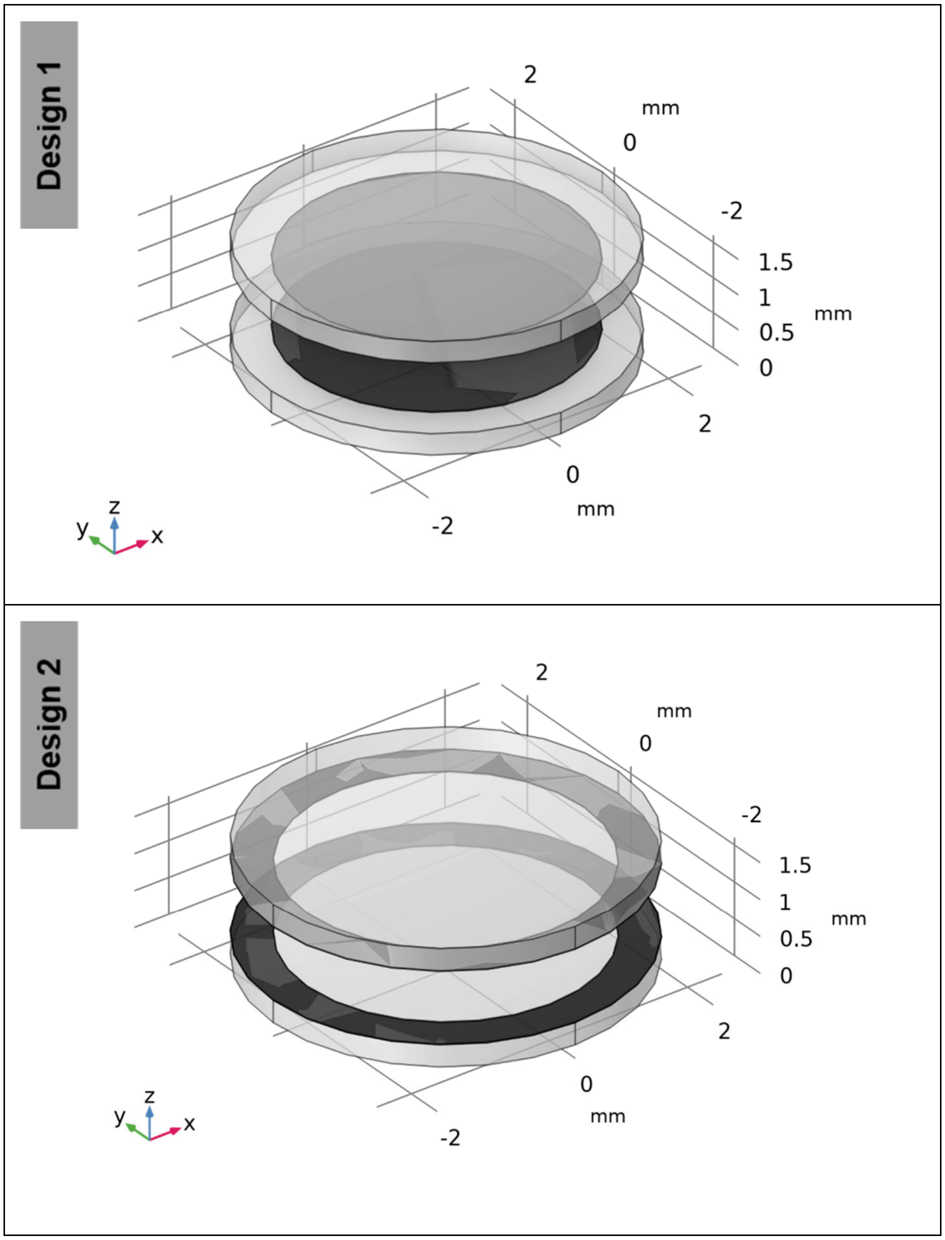

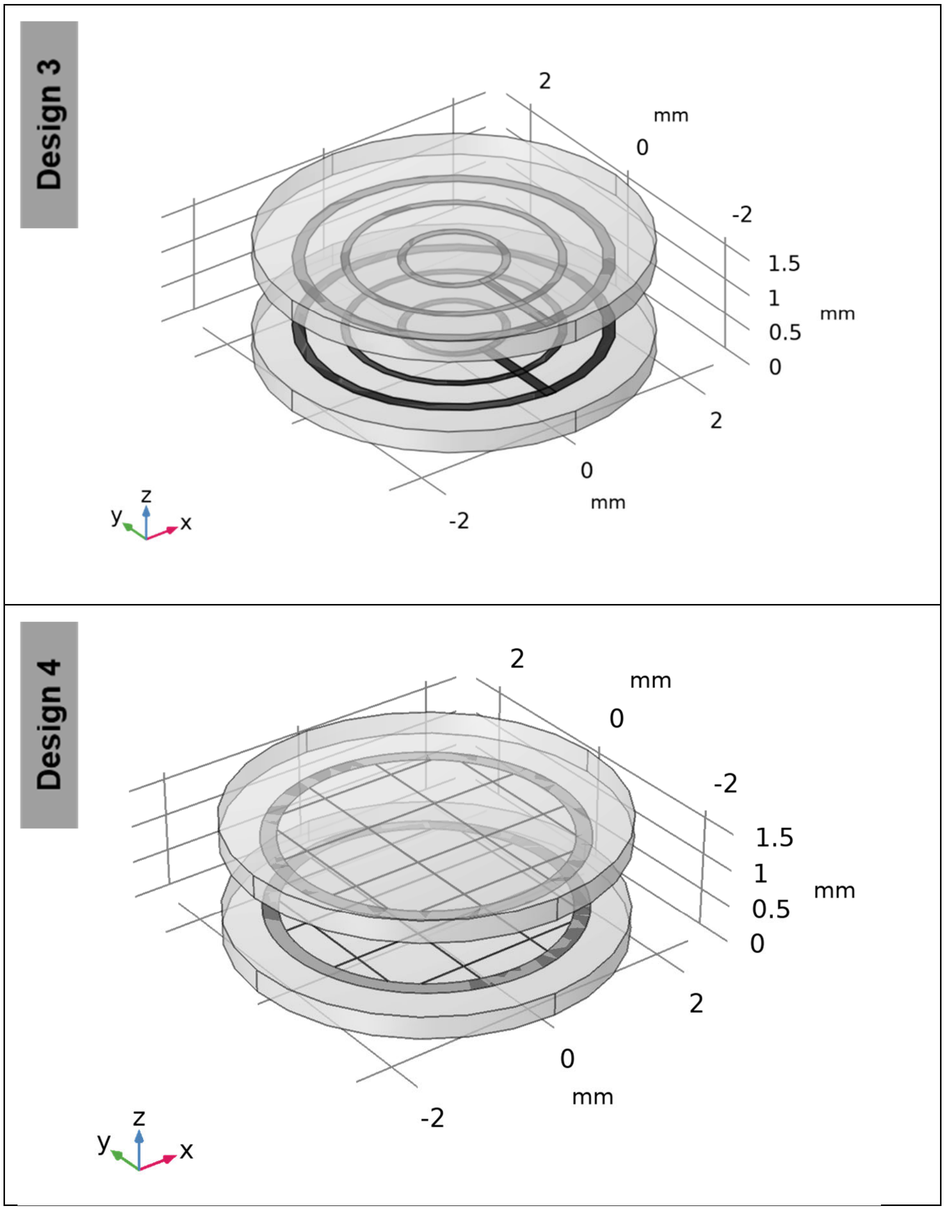

Figure S1. Dimensions and positioning in the cell-culture chamber (in grey) of the different electrodes (in black) used for the design of impedance sensors: (design 1) planar disk-electrodes, (design 2) ring-electrodes, (design 3) multi-ring-electrodes and (design 4) grid-electrodes.

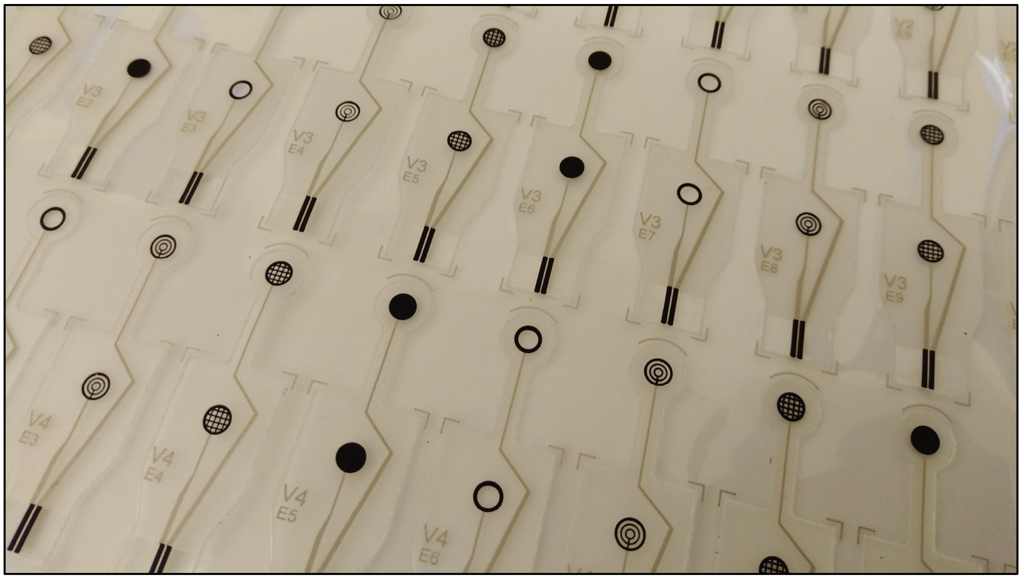

Figure S2. Fabrication process of the electrical impedance spectroscopy sensors using screen-printing technology (fabrication of 40 sensors per batch).

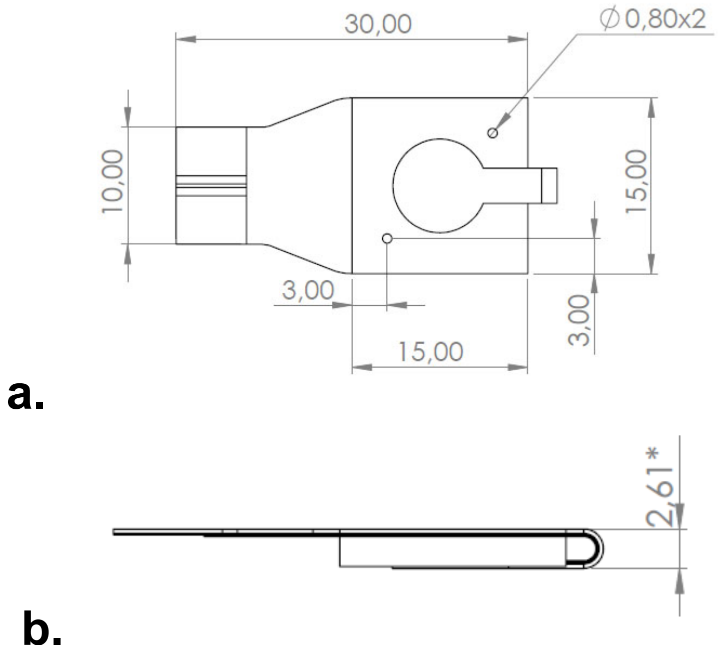

Figure S3. Dimensions of the embedded microfluidic cell-culture chamber (in mm) integrating the impedance sensors in: (**a**) top view and (**b**) side view.

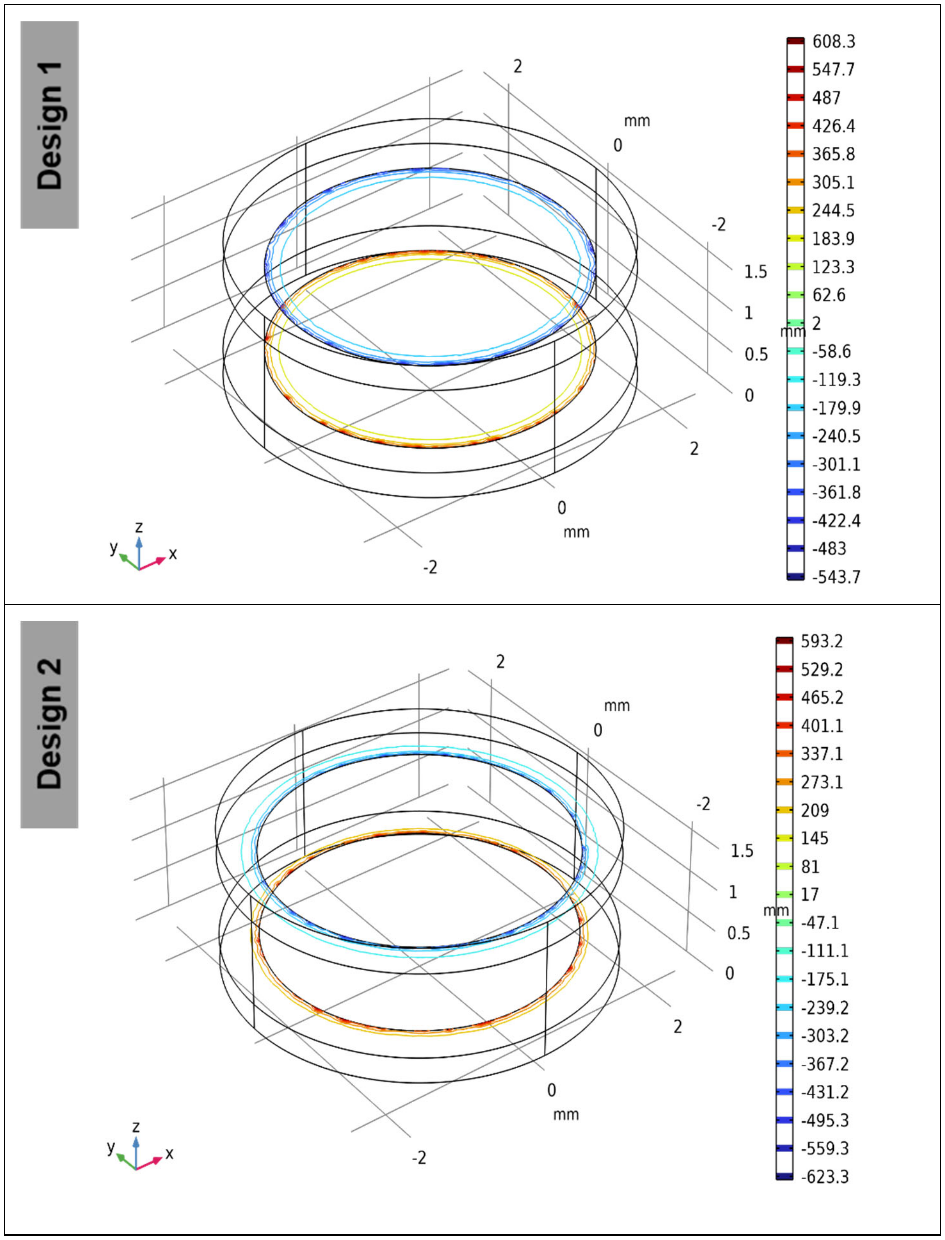

Design 1
608.3
547.7
487
426.4
365.8
305.1
244.5
183.9
123.3
62.6
2
-58.6
-119.3
-179.9
-240.5
-301.1
-361.8
-422.4
-483
-543.7
mm
2
0
-2
1.5
1
0.5
0
x
y
z
Design 2
593.2
529.2
465.2
401.1
337.1
273.1
209
145
81
17
-47.1
-111.1
-175.1
-239.2
-303.2
-367.2
-431.2
-495.3
-559.3
-623.3
mm
2
0
-2
1.5
1
0.5
0
x
y
z

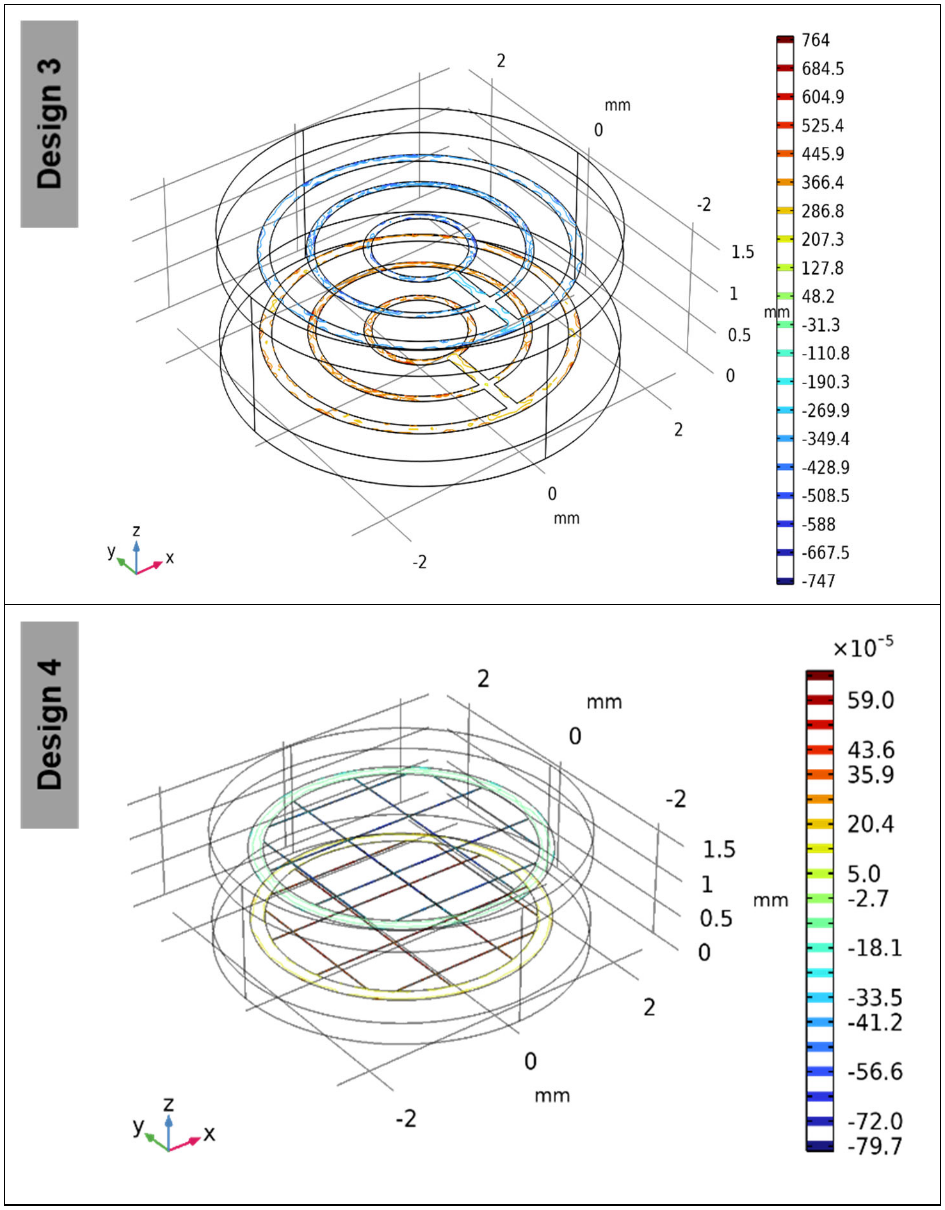

Figure S4. Distribution and variation of the current density (in A/m²) on the electrode surfaces of: (design 1) planar disk-electrodes, (design 2) ring-electrodes, (design 3) multi-ring-electrodes and (design 4) grid-electrodes.

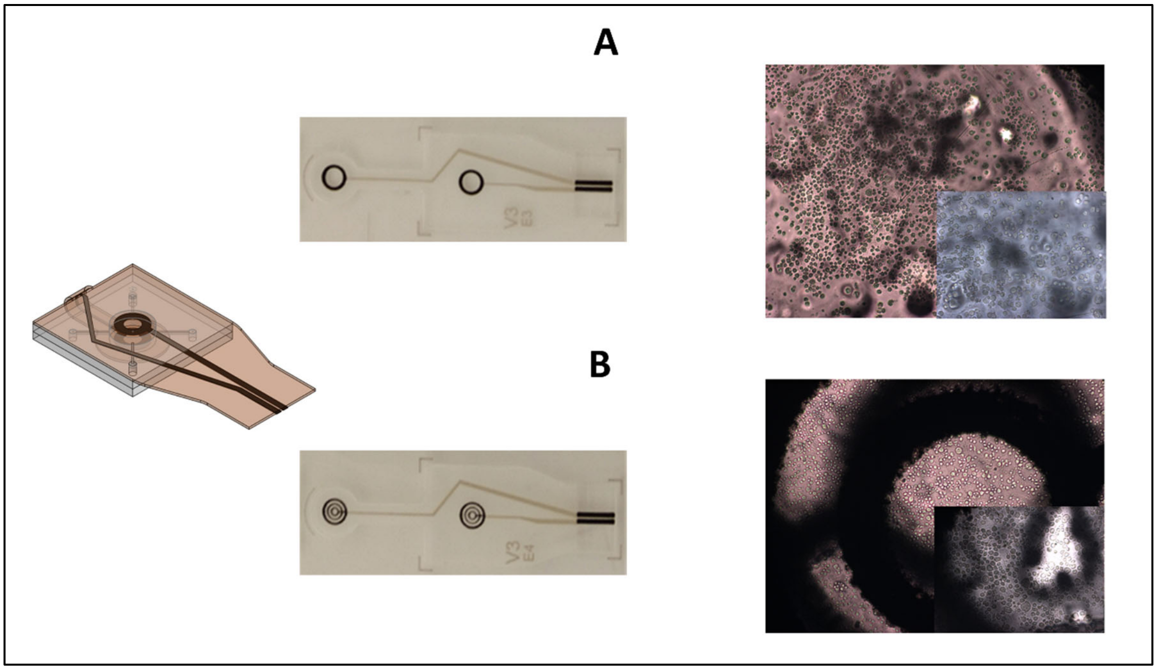

Figure S5. Impedance electrodes and bright field images of Calu-3 cells on (**A**) ring-electrodes design and (**B**) multi-rings electrodes after 4 days in perfused culture; magnification images highlights the cell morphology were Calu-3 cells grown as islands.
